# Tailored ß-Cyclodextrin Blocks the Translocation Pores of Binary Exotoxins from *C. Botulinum* and *C. Perfringens* and Protects Cells from Intoxication

**DOI:** 10.1371/journal.pone.0023927

**Published:** 2011-08-22

**Authors:** Ekaterina M. Nestorovich, Vladimir A. Karginov, Michel R. Popoff, Sergey M. Bezrukov, Holger Barth

**Affiliations:** 1 Department of Biology, The Catholic University of America, Washington, D.C., United States of America; 2 Program in Physical Biology, Eunice Kennedy Shriver National Institute of Child Health and Human Development, National Institutes of Health, Bethesda, Maryland, United States of America; 3 Innovative Biologics, Inc., Herndon, Virginia, United States of America; 4 Department of Host-Pathogen Interactions, Institut Pasteur, Paris, France; 5 Institute of Pharmacology and Toxicology, University of Ulm Medical Center, Ulm, Germany; University of Osnabrueck, Germany

## Abstract

**Background:**

*Clostridium botulinum* C2 toxin and *Clostridium perfringens* iota toxin are binary exotoxins, which ADP-ribosylate actin in the cytosol of mammalian cells and thereby destroy the cytoskeleton. C2 and iota toxin consists of two individual proteins, an enzymatic active (A-) component and a separate receptor binding and translocation (B-) component. The latter forms a complex with the A-component on the surface of target cells and after receptor-mediated endocytosis, it mediates the translocation of the A-component from acidified endosomal vesicles into the cytosol. To this end, the B-components form heptameric pores in endosomal membranes, which serve as translocation channels for the A-components.

**Methodology/Principal Findings:**

Here we demonstrate that a 7-fold symmetrical positively charged ß-cyclodextrin derivative, per-6-S-(3-aminomethyl)benzylthio-ß-cyclodextrin, protects cultured cells from intoxication with C2 and iota toxins in a concentration-dependent manner starting at low micromolar concentrations. We discovered that the compound inhibited the pH-dependent membrane translocation of the A-components of both toxins in intact cells. Consistently, the compound strongly blocked transmembrane channels formed by the B-components of C2 and iota toxin in planar lipid bilayers *in vitro*. With C2 toxin, we consecutively ruled out all other possible inhibitory mechanisms showing that the compound did not interfere with the binding of the toxin to the cells or with the enzyme activity of the A-component.

**Conclusions/Significance:**

The described ß-cyclodextrin derivative was previously identified as one of the most potent inhibitors of the binary lethal toxin of *Bacillus anthracis* both *in vitro* and *in vivo*, implying that it might represent a broad-spectrum inhibitor of binary pore-forming exotoxins from pathogenic bacteria.

## Introduction

Various pathogenic bacteria produce binary exotoxins, which are composed of two non-linked proteins, an enzyme (A-) component and a separate binding and translocation (B-) component. The B-component binds to a receptor on the surface of mammalian target cells, forms a complex with the A-component, and mediates the transport of the A-component into the cytosol, where the enzymatic active A-component modifies its substrate. The binary toxins include lethal toxin and edema toxin from *Bacillus anthracis*, the causative agents of anthrax disease and the family of binary actin-ADP-ribosylating toxins mainly produced by clostridia (for review see [1]). The binary actin-ADP-ribosylating toxins are potent enterotoxins associated with severe human and animal intestinal diseases [2]–[7] and include C2 toxin from *C. botulinum* (types C and D) [3], iota toxin from *C. perfringens* [7], [8], CDT toxin from *C. difficile* [9]–[11] and CST toxin from *C. spiroforme* [12]. The A-components of these toxins mono-ADP-ribosylate G-actin at arginine-177 which turns G-actin into a capping molecule and prevents further polymerization of actin filaments [8], [13], [14]. As a consequence, the actin filaments depolymerise and this results in a complete destruction of the actin cytoskeleton and a rounding up of toxin-treated cells [2], [3], [6], [15]–[18]. Finally, intoxicated cells undergo caspase-dependent cell death [19], [20].

The *C. botulinum* C2 toxin is the prototype of this toxin family (for review see [21]). It consists of the A-component C2I (∼49 kDa) [22], [23] and the B-component C2II (∼80 or 100 kDa, depending on the strain [24], [25]. Following proteolytic activation, C2II forms ring-shaped heptamers (C2IIa) that bind to asparagine-linked carbohydrate structures, which are present on the surface of all the mammalian cell types tested so far [24], [26]–[29]. C2I binds either to receptor-bound C2IIa or to soluble C2IIa prior to receptor-binding [30]. Cell-bound C2IIa/C2I complexes are internalized by receptor-mediated endocytosis [31], [32] and reach the early endosomal vesicles. There, C2I translocates as an unfolded protein across the endosomal membranes into the cytosol and this step is mediated by C2IIa and facilitated by host cell chaperones [24], [33]–[35]. More importantly, the acidification of the endosomal lumen triggers the conversion of C2IIa heptamers into their pore conformation and the insertion of C2IIa pores in endosomal membranes and therefore, pore formation by C2IIa is absolutely essential for translocation of C2I into the cytosol [24], [36]–[39]. In planar lipid bilayer membranes, C2IIa forms ion-permeable, cation-selective and voltage-gated heptameric channels [36]. The C2IIa pores insert into membranes in an oriented manner and are blocked by the addition of C2I to the *cis*-side of the membrane [38]–[39]. It was suggested that C2I interacts with negatively charged residues localized in the pore vestibule [39].

The cellular uptake of the related *C. perfringens* iota toxin, which consists of the A-component Ia and the channel-forming B-component Ib follows a widely comparable mechanism [40] with distinct differences described below (see Discussion). Like C2IIa, activated Ib forms heptameric transmembrane pores *in vitro* [41] and in cell membranes [40], which mediate translocation of Ia into the cytosol.

Novel pharmacological inhibitors of the translocation pores of binary toxins represent attractive candidates to prevent the transport of the A-components into the cytosol and thereby protect cells from intoxication. It was reported earlier that specially designed ß-cyclodextrin derivatives carrying 7-positively charged groups (7+ß-CD) blocked the translocation pore formed by the B-component of anthrax toxins, which is Protective Antigen (PA_63_) [42]–[45]. One compound, per-6-S-(3-aminomethyl)benzylthio-ß-cyclodextrin (AMBnTßCD), efficiently blocked the PA_63_ pores in subnanomolar concentrations on the single molecule level *in vitro* (compound 14b in ref. [43]) and protected cultured macrophage-like cells from intoxication with anthrax lethal toxin (PA+LF), IC_50_ = 0.5±0.2 µM [43]. Most importantly, the compound completely protected Fischer F344 rats [46] from intoxication with lethal toxin and in combination with the antibiotic ciprofloxacin significantly increased the survival of mice in an infection model of anthrax [46], clearly demonstrating its value as a potential drug against anthrax. Several structurally related 7+ß-CDs also protected rabbit erythrocytes against the pore-forming cytotoxic agent of *Staphylococcus aureus*, α-hemolysin [44], [47] and irreversibly blocked heptameric α-hemolysin pores in planar lipid membranes [44]. Here, we demonstrate that AMBnTßCD efficiently protects cultured epithelial cells from intoxication with C2 and iota toxins and blocks the ion current through heptameric channels formed by C2IIa and Ib in planar lipid membranes *in vitro*. This compound was previously identified as one of the most powerful 7+ß-CD inhibitors of anthrax toxins. It is now selected as the most potent blocker of the C2IIa channel by the screening of several 7+ß-CDs with the planar lipid membrane technique. The inhibitory effects of AMBnTßCD were investigated *in vitro* and in intact cells to discover the underlying molecular mechanism. We show that the compound efficiently inhibits the membrane translocation of C2I and Ia into the cytosol of intact cultured cells.

## Results

### The ß-cyclodextrin derivative AMBnTßCD protects mammalian cells from intoxication with the C2 toxin of *C. botulinum*

**Figure 1:**
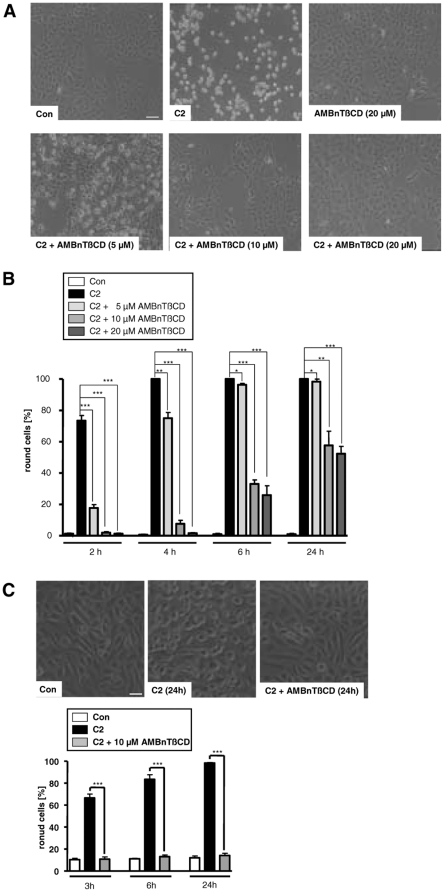
Pre-treatment of Vero epithelial cells and CHO-K1 fibroblasts with the ß-cyclodextrin derivative AMBnTßCD protects cells from intoxication with *C. botulinum* C2 toxin. *A*. Time- and concentration-dependent inhibition of the intoxication of Vero cells by C2 toxin. Vero cells were grown in 24-well dishes to subconfluency and treated with 5, 10 and 20 µM final concentrations of AMBnTßCD for 30 min at 37°C. Subsequently C2 toxin (200 ng/ml C2IIa+100 ng/ml C2I) was added and cells were further incubated with the toxin in the presence of AMBnTßCD at 37°C. For a control, cells were left untreated or treated with C2 toxin alone or with AMBnTßCD alone. Pictures were taken after 2, 4, 6 and 24 h. The morphology of cells is shown after 4 h of C2 toxin-treatment (scale bar = 100 µm) (A). *B*. The number of total cells and round cells were counted from the pictures and the percentages of round cells calculated (lower panel). Values are given as mean ± S.D. (n = 3) and significance was tested for each time point between toxin-treated samples with or without AMBnTßCD by using the student's t-test (***p<0.0005; ***<0.005; *<0.05). *C*. AMBnTßCD inhibits the intoxication of CHO-K1 cells with C2 toxin. CHO-K1 cells were incubated with 10 µM of AMBnTßCD and after 30 min C2 toxin was applied exactly as described above. For a control, cells were treated without toxin or without AMBnTßCD or were left untreated. Pictures from the cells were taken after 3, 6 and 24 h (scale bar = 25 µm) and the percentages of round cells were determined. Values are given as mean ± S.D. (n = 3) and significance was tested for each time point between toxin-treated samples with or without AMBnTßCD by using the student's t-test (***p<0.0005).

To test whether AMBnTßCD has an effect on the intoxication of cells in the presence of C2 toxin, we pre-treated cultured Vero epithelial cells for 30 min with 5, 10 or 20 µM final concentrations of this compound in complete medium and subsequently added the two toxin components C2IIa (200 ng/ml) and C2I (100 ng/ml) to the medium. Cells were further incubated at 37°C and the toxin effect was analyzed in terms of cell rounding, a specific and well-established endpoint to monitor the mode of action of the actin-ADP-ribosylating toxin in the cytosol of mammalian cells. As shown in Fig. 1A, 100% of the cells were round after 4 h of toxin-treatment in the absence of AMBnTßCD while there was no toxin-induced cell rounding in the presence of 10 or 20 µM of AMBnTßCD. In contrast, 5 µM of AMBnTßCD only showed a slight protective effect after a 4 h of incubation with C2 toxin. As expected by the observed changes in cell morphology, the actin was completely ADP-ribosylated in lysates from cells treated with C2 toxin alone while significantly less actin was ADP-ribosylated in the cytosol of cells treated with C2 toxin in the presence of AMBnTßCD (data not shown). To analyze the inhibitory effect of AMBnTßCD on the intoxication of Vero cells with C2 toxin in more detail, we performed a time course and counted the number of total cells and round cells from the pictures to calculate the percentage of round cells for each sample (Fig. 1B). AMBnTßCD inhibited the intoxication of cells with C2 toxin in a time- and concentration-dependent manner and even after a 24 h incubation period there was still a significant (approx. 50%) inhibitory effect in the case of 10 and 20 µM AMBnTßCD (Fig. 1B).

The described inhibitory effect of AMBnTßCD was not restricted to a certain cell-type because we observed a very efficient inhibition of C2 intoxication of CHO-K1 fibroblasts by 10 µM of AMBnTßCD even after 24 h (Fig. 1C). Also, we excluded that the solvent DMSO had any effect on the intoxication of Vero or CHO-K1 cells by C2 toxin (data not shown). Moreover, the treatment of the cells with 10 or 20 µM of AMBnTßCD alone did not change the morphology of Vero cells (Fig. 1A) or CHO-K1 cells, even when the compound was applied to cells for 72 h (data not shown). Therefore, such concentrations of AMBnTßCD were used in the further experiments of this study.

**Figure 2:**
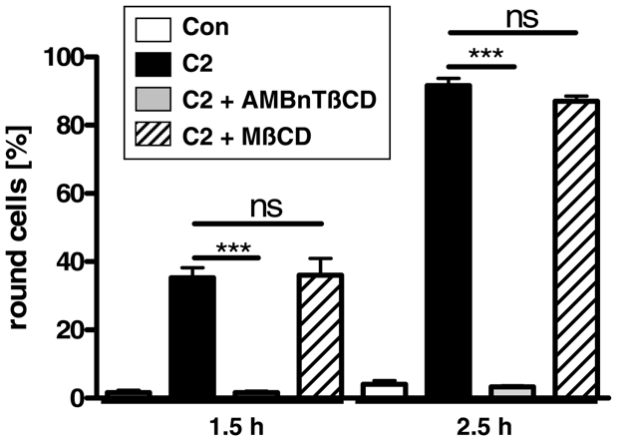
The ß-cyclodextrin derivative AMBnTßCD but not methyl-ß-cyclodextrin protects Vero cells from intoxication with C2 toxin when administered at 10 µM final concentration. Vero cells grown in 24-well plates were treated for 1 h at 37°C with either AMBnTßCD (10 µM) or methyl-ß-cyclodextrin (MßCD, 10 µM) and subsequently cells were challenged with C2 toxin (200 ng/ml C2IIa+100 ng/ml C2I). After 1.5 and 2.5 h of incubation at 37°C, pictures were taken to determine the percentages of round cells. Values are given as mean ± S.D. (n = 3) and significance was tested for each time point between toxin-treated samples and samples treated with either AMBnTßCD plus C2 toxin or MßCD plus C2 toxin by using the student's t-test (***p<0.0005; n. s. = not significant).

Interestingly, the AMBnTßCD but not the structurally related neutral methyl-ß-cyclodextrin (MßCD) inhibited the intoxication of Vero cells with C2 toxin when 10 µM of each ß-cyclodextrin were applied to cells (Fig. 2). In addition, it was reported that much higher (5–10 mM) concentrations of MßCD decreased the binding of the B-components of C2 and iota toxins to the cell surface by removing cholesterol from the cell membrane [31], [48] and protects cells from intoxication with C2 toxin [31]. However, the finding that low micromolar concentrations of MßCD did not affect intoxication with C2 toxin implies that the molecular mechanism underlying the protective effect of AMBnTßCD is specific for this particular compound and not for ß-cyclodextrin molecules *per se* and therefore we investigated this mechanism in more detail. Note that a nonmodified (uncharged) ß-cyclodextrin did not inhibit the cytotoxic effect of *Bacillus anthracis* lethal toxin on the mouse macrophage-like RAW 264.7 cells up to a 100 µM concentrations [42].

**Figure 3:**
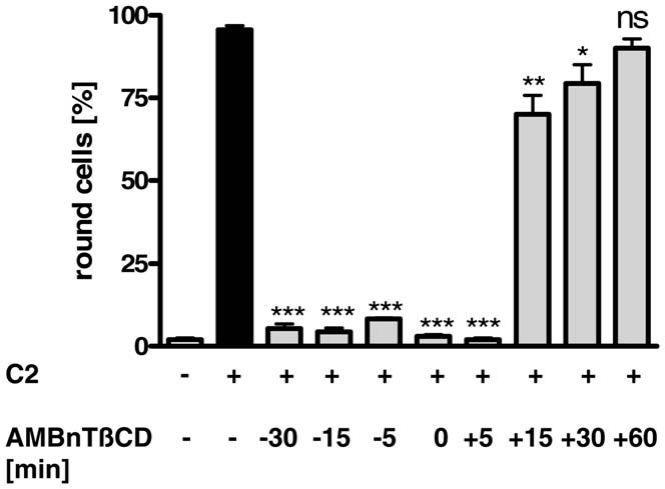
The time point of AMBnTßCD application determines the protective effect of this compound against intoxication of Vero cells with C2 toxin. Vero cells were grown in 24-well plates and AMBnTßCD (10 µM) was applied to the cell medium either 30, 15 or 5 min before C2 toxin (200 ng/ml C2IIa+100 ng/ml C2I) was added to the cells or AMBnTßCD was added together with the toxin into the medium. In parallel, AMBnTßCD was added to the cells 5, 15, 30 or 60 min after the toxin. For a control, cells were treated with medium alone or with C2 toxin in the absence of AMBnTßCD. The cells were incubated for 3 h at 37°C and pictures were taken to determine the percentages of round cells. Values are given as mean ± S.D. (n = 3) and significance was tested for each sample treated with C2 toxin and AMBnTßCD against cells treated with C2 toxin only using the student's t-test (***p<0.0005; **p<0.005; *p<0.05; n. s. = not significant).

**Figure 4:**
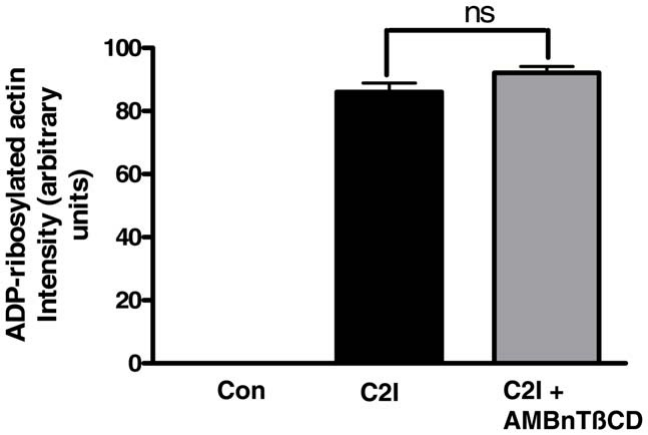
AMBnTßCD does not inhibit the ADP-ribosylation of actin by C2I *in vitro*. Vero lysate (30 µg of protein in 25 µl) was incubated for 10 min at 37°C with 50 ng of C2I and 10 µM biotin-labelled NAD^+^ in the presence or absence of 20 µM P5115. The proteins were separated by SDS-PAGE, blotted onto nitrocellulose and the ADP-ribosylated (i.e. biotin-labelled) actin was detected with streptavidin-peroxidase and a subsequent chemiluminescence reaction. The intensity of bands was determined by densitometry using the Adobe Photoshop 7.0 software.

AMBnTßCD inhibited the intoxication of Vero cells with C2 toxin not only when it was administered to the cells prior to the toxin but also when AMBnTßCD and C2 toxin were applied at the same time point or when AMBnTßCD was added 5 min after C2 toxin application (Fig. 3). In contrast, most cells rounded up after 3 h of toxin-treatment when AMBnTßCD was applied 15 min or more after the toxin. It has been shown, that most of the membrane-bound C2 toxin is internalized by receptor-mediated endocytosis by epithelial cells within 15 min and no more toxin is detectable on the cell surface after 30 min [31]. Therefore, this result is a strong indication that AMBnTßCD interferes with the C2IIa-mediated uptake of C2I into the cytosol of target cells and not with the C2I-catalyzed ADP-ribosylation of actin that occurs when C2I is already delivered into the host cell cytosol. To verify this hypothesis, we tested whether AMBnTßCD has an effect on the enzyme activity of C2I *in vitro* by performing ADP-ribosylation of actin from Vero lysate by C2I in the absence and presence of AMBnTßCD. The data shown in Fig. 4 clearly demonstrate that 10 µM of AMBnTßCD had no effect on the ADP-ribosyltransferase activity of C2I.

### AMBnTßCD inhibits the pH-dependent membrane translocation of C2I through C2IIa pores

**Figure 5:**
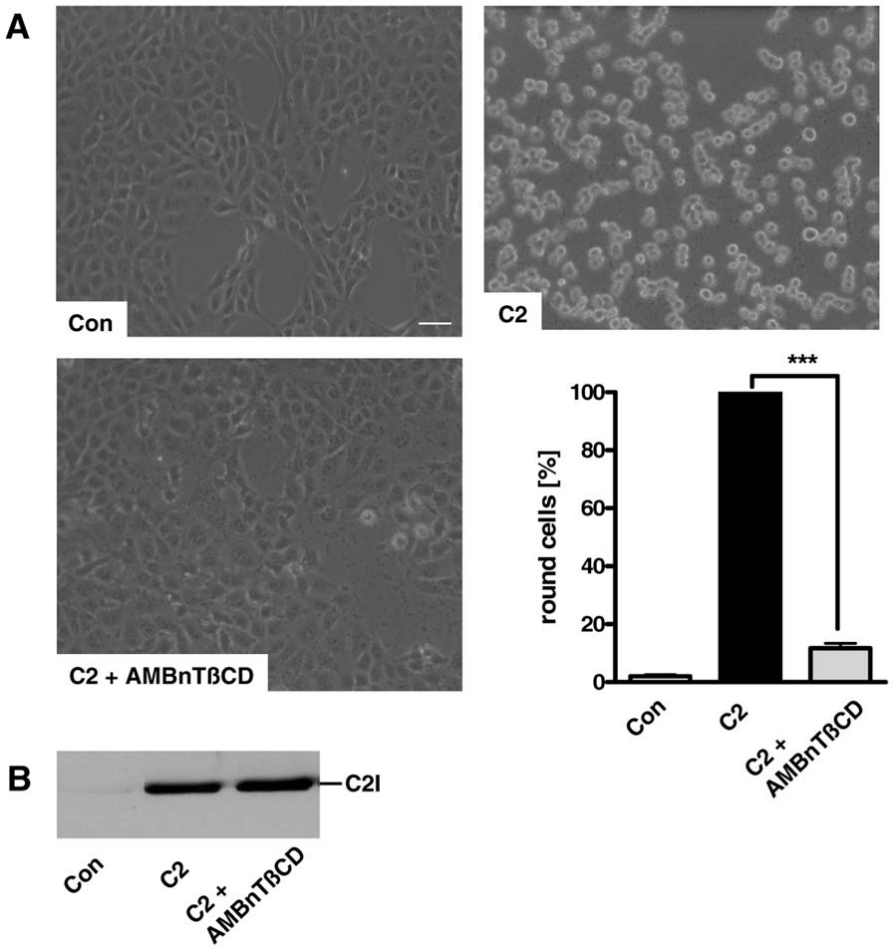
Effect of AMBnTßCD on receptor binding of C2 toxin. Vero cells were incubated for 30 min at 4°C with C2 toxin (200 ng/ml C2IIa+100 ng/ml C2I) to enable toxin binding to the receptor on the cell surface. Then, the medium was removed and cells were washed to remove any unbound toxin. Fresh medium containing 10 µM of AMBnTßCD was added and cells were further incubated at 37°C to trigger internalization of the cell-bound C2 toxin. As a control, cells were incubated with fresh medium without AMBnTßCD or left untreated. After 3 h, pictures were taken to determine the percentages of round cells (scale bar = 100 µm) (*A*). Values are given as mean ± S.D. (n = 3) and significance was tested between toxin-treated samples with or without AMBnTßCD by using the student's t-test (***p<0.0005). *B*. Western blot detection of cell-associated C2I protein. Equal amounts of cell lysate proteins were subjected to SDS-PAGE, blotted and C2I was visualized in a Western blot with a specific antibody against the N-terminal domain of C2I. Purified C2I protein was run as a control in the same gel (not shown).

Having excluded that AMBnTßCD acts on the enzyme activity of C2 toxin, we focused on the molecular mechanism by which AMBnTßCD inhibits the uptake of C2 toxin into the cytosol of the target cells. First, we tested whether AMBnTßCD inhibited the binding of C2 toxin to its receptor on the cell surface. Vero cells were incubated for 30 min at 4°C with C2 toxin to allow the toxin to bind to the receptor. Subsequently, cells were washed to remove unbound C2 toxin and incubated for 3 h at 37°C in toxin-free medium containing 10 µM of AMBnTßCD. For a control, cells were incubated with medium alone. As shown in Fig. 5A, C2 toxin-treated cells were round but the toxin-mediated cell-rounding was inhibited in the presence of AMBnTßCD, strongly suggesting that AMBnTßCD had no effect on the binding of C2 toxin to the cell surface receptor. Consistently, a comparable amount of cell-associated C2I protein was detectable in the lysates from those cells using Western blot analysis with an anti-C2I antibody (Fig. 5B), clearly indicating that AMBnTßCD did not interfere with receptor-binding of C2 toxin. Importantly, equal amounts of cell lysates were analyzed as confirmed by Ponceau S-staining of the blotted proteins (not shown). Moreover, the detected C2I protein specifically bound to cells via the C2IIa component. We have confirmed earlier that in the absence of C2IIa only a negligible amount of C2I was detectable by Western blot analysis [35].

**Figure 6:**
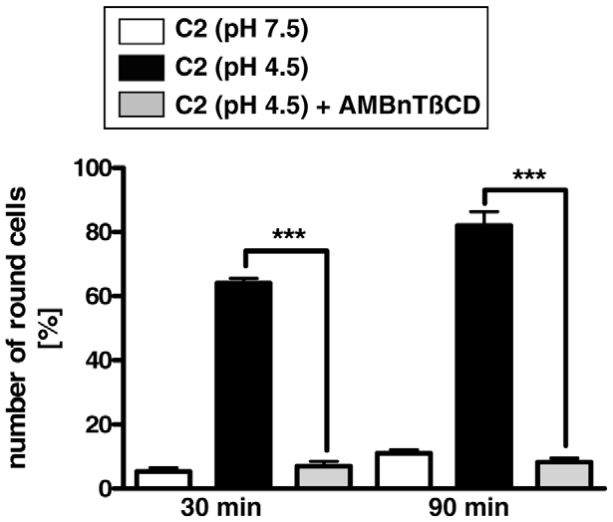
AMBnTßCD inhibits the pH-dependent membrane translocation of C2 toxin across cytoplasmic membranes of intact cells. Vero cells were incubated for 30 min at 37°C with 100 nM Baf A1 and subsequently for 30 min at 4°C in serum-free medium with C2 toxin (400 ng/mL C2IIa+200 ng/ml C2I) or without toxin for control. Then, the medium was removed and cells were exposed to a short acidic shift with warm medium (5 min, pH 4.5, 37°C, Baf A1) to trigger pore formation by C2IIa and membrane translocation of C2I. In parallel, cells were exposed for 5 min to neutral medium (37°C, pH 7.5, Baf A1) as a control. In some samples, AMBnTßCD (10 µM) was present during acidic shift. Subsequently, the medium was changed and cells were further incubated at 37°C in neutral medium, still in the presence of Baf A1 to prevent the normal uptake of C2 toxin via acidified endosomes. Pictures were taken after 30 and 90 min of incubation. The percentages of round (i.e. intoxicated) cells were determined from the pictures, values are given as mean ± S.D. (n = 3). Significance was tested for each time point between samples, treated with C2 under acidic conditions in the absence or presence of AMBnTßCD by using the student's t-test (***p<0.0005).

Therefore, AMBnTßCD most likely inhibits a later step of toxin uptake and thus we next focused on its effect on the pH-dependent translocation of C2I through the lumen of C2IIa pores across cell membranes into the cytosol. We performed a well-established translocation assay, which mimics the endosomal conditions on the surface of intact cells. This assay allows for the monitoring of the direct translocation of C2I through C2IIa pores, which are formed in the plasma membrane under acidic conditions. First, we incubated Vero cells for 30 min at 4°C with C2IIa plus C2I to enable toxin binding to the cells. Then, we exposed the cells for 5 min to warm acidic medium (pH 4.5, 37°C) [49] to trigger membrane insertion and pore-formation by C2IIa and the membrane translocation of C2I through the pores into the cytosol. To test whether AMBnTßCD inhibits membrane translocation of C2I under such conditions, the acidic shift was performed in the absence and the presence of AMBnTßCD. The cells were further incubated at 37°C in neutral medium to allow the ADP-ribosylation of actin by the translocated C2I in the cytosol and after 30 and 90 min of incubation the toxin-induced cell rounding was detected. Also, all the steps of this assay were performed in the presence of Baf A1 to inhibit the physiological uptake of C2 toxin via acidified endosomal vesicles. As shown in Fig. 6, most of the C2 toxin-treated cells were round when they were exposed to the acidic medium in the absence of AMBnTßCD. However, there was no cell rounding when the toxin-treated cells were not exposed to an acidic shift. Moreover, the acidic conditions alone did not cause cell rounding (not shown). Most important, the presence of AMBnTßCD during the acidification step completely prevented C2 toxin-induced cell rounding, even after 90 min, clearly demonstrating that the compound blocked the C2IIa-mediated translocation of C2I across cell membranes.

### AMBnTßCD protects Vero cells from intoxication with iota toxin from *C. perfringens*

**Figure 7:**
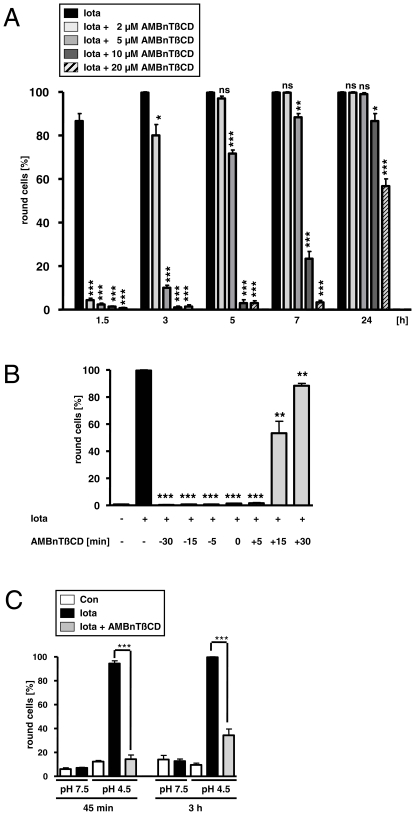
AMBnTßCD protects Vero cells from intoxication with iota toxin from *C. perfringens* and inhibits the pH-dependent membrane translocation of the toxin. *A*. Time- and concentration-dependent inhibition of the intoxication of Vero cells with iota toxin. Vero cells grown in 24-well dishes to subconfluency were treated for 30 min at 37°C with 2, 5, 10 and 20 µM final concentrations of AMBnTßCD or without AMBnTßCD for control. Iota toxin (200 ng/ml Ib+100 ng/ml Ia) was added and cells were further incubated at 37°C with the toxin in the absence or presence of AMBnTßCD. Pictures were taken after the indicated incubation periods, the number of total cells and round cells were counted and the percentages of round cells calculated. Values are given as mean ± S.D. (n = 3) and significance was tested for each time point between iota toxin-treated samples without and with the respective concentration of AMBnTßCD by using the student's t-test (***p<0.0005; ***<0.005; *<0.05). *B*. The time point of AMBnTßCD application determines the protective effect against iota toxin. AMBnTßCD (10 µM) was applied to Vero cells at 30, 15 or 5 min before iota toxin (200 ng/ml Ib+100 ng/ml Ia), together with iota toxin or 5, 15 or 30 min after the toxin. As a control, cells were treated with medium or with iota toxin alone. The cells were incubated for 3 h at 37°C and pictures were taken to determine the percentages of round cells. Values are given as mean ± S.D. (n = 3) and significance was tested between cells treated with iota toxin alone and cells treated with toxin and AMBnTßCD by using the student's t-test (***p<0.0005; **p<0.005). *C*. AMBnTßCD inhibits the pH-dependent membrane translocation of iota toxin across the cytoplasmic membranes of intact Vero cells. Cells were incubated for 30 min at 37°C with 100 nM Baf A1 and subsequently for 30 min at 4°C in serum-free medium with iota toxin (1000 ng/mL Ib+500 ng/ml Ia) or without toxin for control. Then, 10 µM of AMBnTßCD were added (for control no AMBnTßCD) and the pH of the medium was adjusted to 4.5 with HCl (for control pH 7.5) and cells were exposed for 15 min to 37°C to trigger pore formation by Ib membrane translocation of Ia. Subsequently, cells were further incubated at 37°C in neutral medium containing Baf A1 and pictures were taken after 45 min and 3 h of incubation. The percentages of round (i. e. intoxicated) cells were determined, values are given as mean ± S.D. (n = 3). Significance was tested for each time point between samples treated with iota toxin under acidic condition in the absence or presence of AMBnTßCD by using the student's t-test (***p<0.0005).

Prompted by the results obtained for C2 toxin, we finally investigated whether AMBnTßCD blocks the translocation pores of the binary iota toxin, likewise. We found that AMBnTßCD inhibited the intoxication of Vero cells with iota toxin. Cells were pre-treated for 30 min with various concentrations of AMBnTßCD before iota toxin was added to the medium. The presence of AMBnTßCD protected cells from intoxication with iota toxin in a time- and concentration-dependent manner as shown in Fig. 7A. Concentrations of 10 and 20 µM of AMBnTßCD had a partial but significant protective effect for up to 24 h. As we have observed before for C2 toxin, AMBnTßCD exhibited its full protective effect when it was applied before, simultaneously or even shortly after the toxin, but there was much less protection when AMBnTßCD was applied 15 or 30 min after iota toxin (Fig. 7B), implying that AMBnTßCD had no inhibitory effect after the enzyme component of the toxin reached the cytosol. Consistent with our results obtained for C2 toxin, AMBnTßCD inhibited the pH-induced membrane translocation of iota toxin (Fig. 7C), implying that this cyclodextrin derivative interferes with the same molecular mechanisms during cellular uptake of the binary C2 and iota toxins. Moreover, iota toxin represents the prototype of the group of closely related iota-like toxins and therefore, these findings strongly suggest that AMBnTßCD might be a universal inhibitor for the complete family of binary actin ADP-ribosylating toxins from pathogenic clostridia.

### AMBnTßCD blocks C2IIa and Ib Channels in Planar Lipid Bilayers

**Figure 8:**
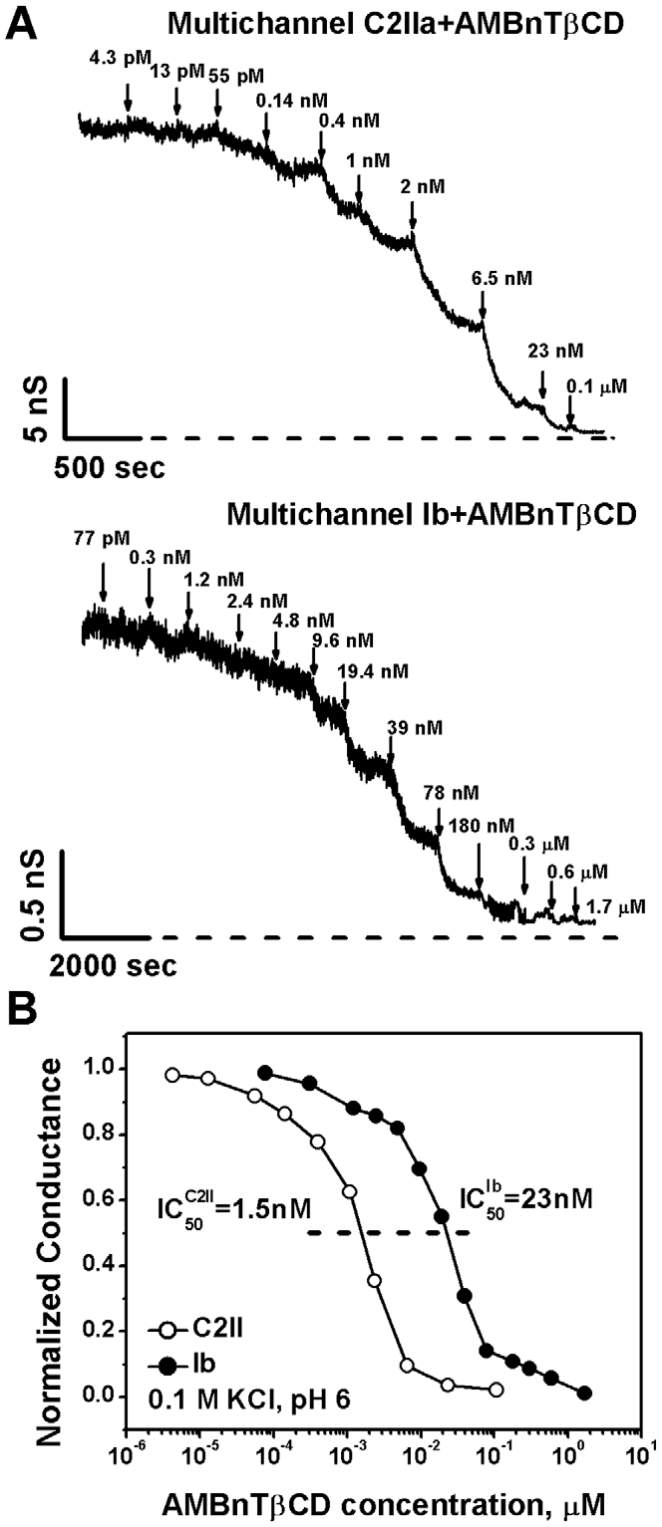
AMBnTßCD blocks the transmembrane pores formed by C2IIa and Ib in planar lipid membranes at the multichannel level. *A*. Multichannel C2II (top) and Ib (bottom)-induced conductance changed by AMBnTßCD addition. The current recordings were additionally filtered over 500-ms time interval. 0.1 M KCl solutions at pH 6 were buffered by MES. Recordings were taken at 20 mV applied voltage. *B*. Typical multichannel titration curves for C2IIa (left) and Ib (right)-modified membranes show about 15 times lower IC_50_ values for C2II channels. All multichannel measurements were taken at 20 mV applied voltage.

AMBnTßCD and structurally related positively charged ßCDs were recently reported to electrostatically interact with the negative charges inside the lumen of the PA_63_ channel of anthrax [42], [43], [45] directly blocking the translocation pathway of the pore. To find out if the mechanism of AMBnTßCD-induced inhibition of the channel-forming components of clostridial binary toxins is identical to that of *Bacillus anthracis* toxins, we first reconstituted C2IIa and Ib pores into planar lipid bilayers and studied the effects of AMBnTßCD on the multichannel membranes in 0.1 M KCl (Fig. 8). We found a profound inhibition of both C2IIa (Fig. 8, top) and Ib (Fig. 8, bottom) ion current at nM – low µM concentrations of the blocker (Fig. 8B).

**Figure 9:**
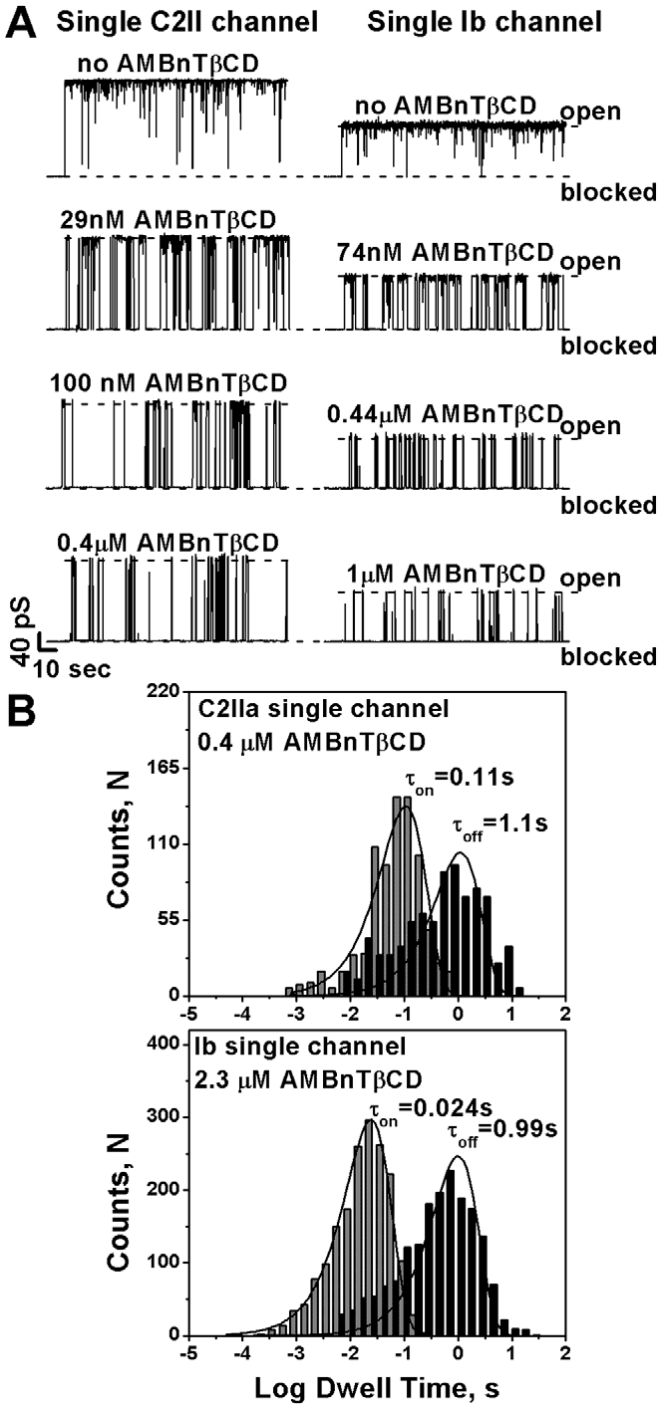
AMBnTßCD blocks the single pores formed by C2IIa and Ib in planar lipid membranes. *A*. Currents through single C2IIa (left) and Ib (right) channels at 50 mV applied voltage. The two topmost tracks represent the AMBnTßCD-free control experiments. The fast flickering between open and closed states (1/f noise) was mostly but not completely removed here by averaging over 100-ms time interval. In the presence of increasing AMBnTßCD, the channels were spontaneously blocked (three lower current tracks). The dashed lines represent zero current levels. *B*. Typical time histograms of AMBnTßCD-induced C2IIa (top) and Ib (bottom) current fluctuations. Original current recordings collected with 15 kHz filter and 50 kHz sampling were additionally filtered with a 300 Hz filter to exclude most of the high-frequency 1/f events. T_off_ represents the time channels spent in the blocked state and T_on_ is the time between successful blockages. Data were fitted by direct single-exponential (i.e. log probability) fitting [79]. The fits were obtained using variable metrics as a search method and maximum likelihood as a minimization method. Measurements were performed in 1 M KCl solutions at pH 6 and 50 mV applied voltage. 0.4 µM and 2.3 µM AMBnTßCD concentrations were used for the C2IIa and Ib time-histograms, respectively, as statistically more represented.

To study the kinetics of channel-blocker interactions on a single-molecule/single-channel level, we switched to 1 M KCl concentrations. Higher salt concentrations produce a higher signal to noise ratio in single-channel measurements. Moreover, the statistically reliable quantitative analysis of single-channel blockage turned out to be difficult at physiological salt concentrations because the residence time of AMBnTßCD in the channel was very long (minutes). The typical recordings of the ion channel through a single C2IIA and a single Ib pore in 1 M KCl are shown in Fig. 9A (left and right respectively). Single-channel conductances of (150±10) pS and (90±5) pS (50 mV) for C2IIa and Ib are in excellent agreement with the results of the pioneering studies performed by Roland Benz's group [36], [41]. We never observed the two types of single-channel insertions for C2IIa and Ib oligomers in contrast to PA_63_ where two types of insertions with slightly different conductance and current noise characteristics were routinely detected [45], [50]. Interestingly, the two types of non-Markov channel gatings previously reported [51]–[55] and recently studied on a single-channel level [45] for the PA_63_ channel were also observed with C2IIa and Ib single channels. The first type of PA_63_-like gating is promoted by applied transmembrane voltage [36], [39], [41] and resembles the voltage-induced closure observed in many known β-barrel channel-forming proteins reconstituted into lipid bilayers ([56] and see related discussion in [39]). Like PA_63_, both C2IIa and Ib possess voltage-independent fast-flickering between the open and completely closed states with the current noise power spectra containing a 1/f-like component (data not shown). This finding agrees with the multichannel experiments [57] where 1/f noise for the C2IIa channels has also been reported; similar switches between the open and closed states of the Ib individual pores were also mentioned before [41]. The current flickering corresponding to 1/f noise was filtered here by averaging over a time interval of 100 ms. The longest 1/f events can still be seen in the absence of AMBnTßCD (Fig. 9A, top current tracks).

In the presence of AMBnTßCD on the *cis* side of the chamber, both channels get spontaneously blocked with the frequency of blockages depending on AMBnTßCD concentration (Fig. 9A, three lower tracks). Within the accuracy of our measurements, these blockages are complete. As with the PA_63_ pore, current fluctuations induced by AMBnTßCD at 1 M KCl can be described as a two-state Markov process, one state being an open pore and a second state being a pore occupied by one blocker molecule. This is manifested by single-exponential distributions both of the duration of AMBnTßCD blockages, T_off_ and the channel lifetime in the unblocked state (the time between successful blockages), T_on_ (Fig. 9B).

## Discussion

Here we have performed a series of *in vitro* and cell-based experiments to demonstrate that the low molecular weight compound AMBnTßCD, a positively charged tailor-made ß-cyclodextrin derivative, is an efficient pharmacological inhibitor for the clostridial C2 and iota toxins. We showed that the compound docks to the transmembrane pores formed by the B-components C2IIa and Ib in planar lipid bilayer membranes and thereby blocks the ion-permeable channels *in vitro*. Consistent with this observation, we found that AMBnTßCD inhibits the pH-dependent translocation of the A-components C2I and Ia through the translocation pores C2IIa and Ib, respectively, across the cytoplasmic membranes of intact cultured epithelial cells. Thus, the data strongly suggests that AMBnTßCD blocks the translocation pores of the toxins in endosomal membranes thereby preventing translocation of the A-components from the endosomal lumen into the cytosol of target cells. Because AMBnTßCD did not interfere with the earlier steps of toxin uptake or the toxin-catalyzed ADP-ribosylation of actin, the data imply that the blocking of the translocation pores is the molecular mechanism underlying the observed protective effects of this compound. Note that fluorescent microscopy data [58] indicate that βCD might also bind to the PA_63_ precursor – the receptor-bound heptameric prepore - before endocytosis takes place. We cannot completely exclude that similar binding occurs between AMBnTßCD and C2IIa and Ib prepores.

### Cellular uptake of the binary C2 and iota toxins as compared with *Bacillus anthracis* toxins

Although C2 toxin and iota toxin belong to the same family of binary actin-ADP-ribosylating toxins and share a widely comparable cellular uptake mechanism, there are some remarkable differences between both toxins [29], [41], [48], [59]. Iota toxin represents the prototype of the iota-like toxins, which include CDT toxin from *C. difficile*, and CST toxin from *C. spiroforme* (for review see ref 1). The B-components of these toxins form biologically active toxin complexes with the A-component of any other member of this group but not with C2 toxin. C2IIa binds to a carbohydrate receptor [27], which is ubiquitously present on mammalian cells and therefore, all cell types tested so far are sensitive towards C2 toxin. In contrast, Ib binds to an as yet unidentified protein receptor [60] and some mammalian cell lines are relatively resistant towards iota toxin. Also, the A-component of C2 toxin translocates from early endosomes into the cytosol, while the A-component of iota toxin most likely escapes from acidified vesicles between early and late endosomes [59] and in contrast to C2 toxin, iota toxin requires a membrane potential gradient in addition to a pH-gradient across the endosomal membrane [59].

As with binary clostridial toxins, the channel-forming B-component (PA_63_) of *Bacillus anthracis* toxins mediates the translocation of the A-components from endosomal vesicles into the cytosol (for details see ref. [61], [62]). The enzyme components are lethal factor (LF, 90 kDa), a metalloprotease that cleaves MAP kinase kinases and induces the cell death of macrophages [63]–[65] and edema factor (EF, 89 kDa), which is an adenylyl cyclase [66], [67]. At mildly acidic conditions, C2IIa, Ib and PA_63_ form ion-permeable and cation-selective oligomeric channels in planar lipid membranes [36], [37], [41], [50]–[55], [57], [68] with a single tunnel inside. PA_63_ strongly prefers cations over anions with a permeability ratio of 20∶1 [51]. 10∶1 and 6∶1 cation to anion ratios were also reported for C2IIa and Ib channels, respectively [36], [41]. C2IIa and Ib are most likely heptamers and PA_63_ was reported to form octamers as well as heptamers [50], [69], [70]. The ability of small molecules carrying one or two positive charges to block an ion current through the PA_63_, C2IIa, and Ib channels was shown earlier [37], [41], [52]–[54], [71], [72]. One of the compounds, positively charged chloroquine not only inhibited C2IIa pores *in vitro* (K_d_ = 10 µM in 0.15 M KCl) [72], but also prevented translocation of C2I across the cell membrane when studied with intact cultured cells [37]. At the same time, only a weak interaction was reported for chloroquine (K_d_ = 0.22 mM in 0.1 M KCl) with Ib channels and chloroquine did not efficiently protect cells from intoxication with iota toxin [41].

### AMBnTßCD effectively inhibits C2, iota, and anthrax toxins

Recently Karginov et al. exploited the well-known information of the molecular structure of PA_63_ prepores for the structure-based design of tailored ß-cyclodextrin derivatives [42], [43] with AMBnTßCD being one of the most active compounds against anthrax lethal toxin [43], [46]. Here we discovered an even broader applicability of AMBnTßCD. It inhibits the pH-dependent membrane translocation of the A-components of clostridial C2 and iota toxins by blocking the transmembrane channels formed by the B-components of these toxins. The question arises: is the mechanism of AMBnTßCD-induced blockage exactly the same for all three pores?

In 0.1 M KCl concentrations, AMBnTßCD blocks ion current through multichannel membranes with affinity decreasing in order IC_50_(PA_63_) = 0.1 nM, IC_50_(C2IIa) = 1.5 nM, and IC_50_(Ib) = 23 nM ([43] and Fig. 8B). These numbers are influenced by a recently described 2^nd^ mode of 7+ß-CD-induced action – blocker-enhanced voltage gating that is much more pronounced at lower salt concentrations [45]. This effect was also observed with C2IIa and Ib channels (data not shown). Note that the less voltage-sensitive Ib pore is only weakly affected by this 2^nd^ mode of AMBnTßCD action. Since the physiological importance of voltage gating is questionable, it is important to compare the binding constants for the 1^st^, equilibrium mode of AMBnTßCD action. This is only possible in single-channel measurements at 1 M KCl where long-lasting voltage gating events can be excluded. The average residence times of the AMBnTßCD compound in the pores, T_off_(C2IIa) = (1.1±0.28) s and T_off_(Ib) = (0.96±0.19) s, (Fig. 9B) are very close to the time reported earlier (1.15±0.2) s for the blocking of the PA_63_ channel by AMBnTßCD under identical conditions (1 M KCl, −50 mV) [43]. This is a very interesting observation taking into account that structurally related 7+ß-CDs often demonstrate quite distinct binding off-rates in case ofPA_63_ channels [43]. Similar behavior is found for C2IIa channels (data not shown). For instance with PA_63_, a 200 times difference in T_off_ values was reported earlier for different 7+ß-CDs [43], [44]. Moreover, the on-rate constants k_on_ = 1/(t_open_c_bl_), where c_bl_ is the bulk AMBnTßCD concentration, equal to (2.3±0.5)×10^7^ (Ms)^−1^ (C2IIa) and (1.8±0.3)×10^7^ (Ms)^−1^ (Ib) which is comparable with the on-rate constants reported for PA_63_-7+β-CD interactions, k_on_ = (2.3±0.6)×10^7^ (Ms)^−1^ [45]. The similarity between the k_on_ values, that are mostly determined by the radius of the channel entrance, may be attributed to the structural resemblance between these heptameric pores (at least at the endosomal cap sides). The dissociation constants K_D_ = k_off_/k_on_ in 1 M KCl solutions are also close K_D_(C2IIa) = 4×10^−8^ M, and K_D_(Ib) = 5.6×10^−8^ M. This similarity strongly suggests close mechanisms of AMBnTßCD-induced pore blockage implying that similar sets of negatively charged amino-acid residues are involved in the interaction with the blocker.

In recent detailed study [45] it was shown that structurally related per-6-(3-aminopropylthio)-β-cyclodextrin (AmPrßCD) blocks PA_63_ pores without translocating through. The binding site for AmPrßCD was estimated to be approximately at 15% from the channel *cis*-entrance suggesting interaction between seven positive charges on the blocker and seven negative charges in the channel cap [42], [45]. On the other hand, about one order of magnitude increase in 7+ß-CD binding affinity was achieved as a result of introducing aromatic (Bn) groups into the alkyl spacers of the 7+ß-CD derivatives, [43], [46]. The specific hydrophobic interactions inside PA_63_ (especially for aromatic compounds) were attributed to the existence of the ϕ-clamp formed by a ring of seven solvent-exposed aromatic phenylalanine groups, Phe-427 [71]. It is tempting to suggest that the hydrophobic interaction of the aromatic groups of AMBnTßCD with the ϕ-clamps contributes significantly to the overall blocker-pore interactions. Note that the Phe at the corresponding position is conserved in both C2IIa and Ib [72], but the importance of the ϕ-clamp for the iota toxin transport is not clear so far. It is not obvious, however, if AMBnTßCD, which has a diameter of about 27 Å, can slip deep enough into the channel to be able to interact with the ϕ-clamp located at 40% of the channel length. More detailed single-channel studies of channel-blocker interaction are currently under way. Recent developments in 7+ß-CDs chemistry and synthesis [73], and nanopore applications [74] provide growing possibilities for these studies.

Taken all together, AMBnTßCD effectively blocks the translocation of the A-components by blocking the pores formed by B-components both *in vitro* and in cell assays for at least three binary pore-forming toxins: Clostridial C2 and iota as well as *Bacillus anthracis* toxins. The protective effect of AMBnTßCD against anthrax lethal toxin in mice was already demonstrated [42] and next steps will include animal studies to test whether AMBnTßCD prevents the adverse effects caused by C2 and iota toxins *in vivo*. Most pertinent, a protective effect of this compound towards the toxin-induced diarrhea could be investigated in the mouse intestinal loop assay [4]. In conclusion, the compound AMBnTßCD might serve as a universal pharmacological inhibitor against binary pore-forming toxins produced by pathogenic bacteria and, therefore, represents a highly attractive potential drug candidate.

## Materials and Methods

### Materials and Reagents

Cell culture medium DMEM and fetal calf serum were obtained from Invitrogen (Karlsruhe, Germany) and HAM's F12 from GIBCO (Karlsruhe, Germany). Cell culture materials were obtained from TPP (Trasadingen, Switzerland). The recombinant C2 toxin components C2I and C2IIa were prepared and activated as described earlier [23], [24]. The components Ia and Ib from the iota toxin were prepared as described earlier [75]. The following chemical reagents were used: KCl, MES, KOH, and HCl (Sigma-Aldrich, USA); “purum” hexadecane (Fluka, Buchs, Switzerland); diphytanoyl phosphatidylcholine (Avanti Polar lipids, Inc., Alabaster, AL); pentane (Burdick and Jackson, Muskegon, MI), agarose (Bethesda Research Laboratory, Gaithersburg, MD). Doubly distilled and deionized water was used to prepare solutions. All solutions were purified by filtration through a 0.45 µm filter. AMBnTßCD was custom synthesized at CycloLab (Budapest, Hungary) with the details of the synthesis given earlier (compound 14b, ref [43]). For planar lipid membrane measurements, Low Binding Polymer Technology products were used for cyclodextrin storage, dilution, and sampling (Sorenson BioScience, Inc., Salt Lake City, UT). Methyl-ß-cyclodextrin was acquired from Sigma-Aldrich (Munich, Germany). Complete® protease inhibitor was purchased from Roche (Mannheim, Germany). Ponceau S was from AppliChem (Weiterstadt, Germany). The protein molecular weight marker Page Ruler prestained Protein ladder® was from Fermentas (St. Leon-Rot, Germany). Biotinylated NAD^+^ was supplied by R&D Systems GmbH (Wiesbaden-Nordenstadt, Germany). Baf A1 was obtained from Calbiochem (Bad Soden, Germany). The enhanced chemiluminescence (ECL) system was from Millipore (Schwalbach, Germany).

### Cell culture and intoxication assays

Vero (African green monkey kidney) cells and CHO-K1 (Chinese hamster ovary) cells were from DSMZ (Braunschweig, Germany). Vero cells were cultivated in MEM containing 10% heat-inactivated fetal calf serum, 1.5 g/L sodium bicarbonate, 1 mM sodium-pyruvate, 2 mM L glutamine and 0.1 mM non-essential amino acids. CHO-K1 cells were cultivated in DMEM and HAM's F12 containing 5% heat-inactivated fetal calf serum, 1 mM sodium-pyruvate and Penicillin-Streptomycin (1∶100). The cell lines were cultivated at 37°C and 5% CO_2_. Cells were trypsinized and reseeded 15–20 times at most. For cytotoxicity experiments, cells were seeded in culture dishes and incubated in serum-free medium with the respective toxin. The cells were incubated with C2 toxin or iota toxin in the absence or presence of the inhibitor AMBnTßCD in complete medium at 37°C, as indicated in detail in the individual legends for the experiments. After the given incubation periods, the cells were visualized by using a Zeiss Axiovert 40CFl microscope (Oberkochen, Germany) with a Jenoptik progress C10 CCD camera (Jena, Germany). The cytopathic effects caused by the toxins were analysed in terms of morphological changes, i.e. cell rounding.

### Toxin translocation assay with intact cells

The pH-dependent translocation of C2I through C2IIa pores across endosomal membranes was experimentally mimicked on the surface of intact cultured cells as described in detail earlier [24]. In brief, Vero cells were incubated with C2IIa/C2I at 4°C to enable the binding of the toxin to the cell surface but to prevent toxin internalization. Then, the cells were exposed to a short acidic pulse (medium with pH 4.0) at 37°C to trigger the pore formation of C2IIa into cell membranes and the translocation of C2I through the pores across the cytoplasmic membrane into the cytosol. It is important to note that bafilomycin A1 was present in the medium during all of the steps to prevent the physiological uptake of the toxin via acidified endosomal vesicles. The toxin-induced cell rounding was monitored and documented to demonstrate that C2I was indeed in the cytosol. The pH-dependent translocation of Iota toxin across the cytoplasmic membranes of Vero cells was performed mainly as described earlier [40].

### Sequential ADP-ribosylation of actin in lysates from toxin-treated cells

For ADP-ribosylation of actin in a cell-free system, 30 µg of whole Vero lysate protein were incubated for 60 min at 37°C in a buffer containing 20 mM Tris-HCl (pH 7.5), 1 mM EDTA, 1 mM DTT, 5 mM MgCl_2_, complete® protease inhibitor, together with biotin-labelled NAD^+^ (10 µM) and 10 or 50 ng of C2I protein. The reaction was stopped with 5×SDS-sample buffer (625 mM Tris/HCl pH 6.8, 20% SDS, 8.5% glycerol, 0.2% bromphenol blue, 100 mM DTT) and the heating of the samples for 5 min at 95°C. The samples were subjected to SDS-PAGE, transferred to a nitrocellulose membrane and the biotin-labelled ADP-ribosylated actin was detected with peroxidase-coupled streptavidin and a subsequent chemiluminescence reaction. Intensity of the biotin-labelled actin was determined by densitometry using the Adobe Photoshop 7.0 software.

### ADP-ribosylation of actin by C2I in a cell-free system

Vero lysate (30 µg of protein) was incubated for 10 min at 37°C in a volume of 25 µl with 50 ng of C2I and 10 µM biotin-labelled NAD^+^ in the presence or absence of AMBnTßCD (20 µM final concentration). Samples were subjected to SDS-PAGE and blotted onto a nitrocellulose membrane. ADP-ribosylated, i.e. biotin-labelled, actin was detected with streptavidin-peroxidase and the ECL system. The intensity of the biotin-labelled actin was determined by densitometry using the Adobe Photoshop 7.0 software.

### SDS-PAGE and immunoblot analysis

For immunoblot analysis of cell-associated C2I, equal amounts of cell lysate protein were subjected to SDS-PAGE according to the Laemmli method [76]. Subsequently, the proteins were transferred to a nitrocellulose membrane (Whatman, Dassel, Germany) what was confirmed by staining of the blotted proteins with Ponceau S. The membrane was blocked for 30 min at room temperature with 5% non-fat dry milk in PBS containing 0.1% Tween-20 (PBS-T). To detect C2I, the membrane was probed with a specific antibody raised against the N-terminal domain of C2I (produced in rabbits by Pineda, Berlin, Germany). After washing with PBS-T, the membrane was incubated with an anti-rabbit antibody coupled to horseradish peroxidase (Santa-Cruz, Heidelberg, Germany), and then washed again. C2I was visualized using the ECL system according to the manufacturer's instructions.

### Channel Reconstitution into Planar Lipid Bilayers

To form solvent-free planar lipid bilayers with the lipid monolayer opposition technique [77], we used a 5 mg/mL stock solution of diphytanoyl phosphatidylcholine in pentane. Bilayer lipid membranes were formed on a 60-µm (for single-channel measurements) or 150-µm (for multichannel measurements) diameter aperture in the 15-µm-thick Teflon film that separated the two compartments as described in detail before [78]. The 0.1 M and 1 M aqueous solutions of KCl were buffered at pH 6 (MES) at room temperature (23±0.5)°C. We also performed several control measurements in MES-free solutions at pH 6. Single channels were formed by adding (0.2–0.5) µl of 48 ng ml^−1^ solution of C2IIa or (0.2–1) µl of 2.5 µg ml^−1^ solution of Ib to the 1.5 ml aqueous phase on the *cis* half of the chamber. For multichannel experiments, we applied about 1–2 µl of 48 µg ml^−1^ C2IIa or 2–3 µl of 2.5 mg ml^−1^ Ib stock solutions to the *cis*-side of the membrane. Under this protocol, C2IIa and Ib channel insertions were always directional as judged by channel conductance asymmetry in the applied transmembrane voltage. The electrical potential difference across the lipid bilayer was applied with a pair of Ag-AgCl electrodes in 2 M KCl, 1.5% agarose bridges. Multichannel measurements were performed at 20 mV and single-channel measurements at 50 mV. The applied potential is defined as positive if it is higher on the side of protein addition (*cis*-side).

Conductance measurements were done using an Axopatch 200B amplifier (Axon Instruments, Inc., Foster City, CA) in the voltage clamp mode. Signals were filtered by a low-pass 8-pole Butterworth filter (Model 9002, Frequency Devices, Inc., Haverhill, MA) at 15 Hz for multichannel and 15 kHz for single-channel systems and sampled with a frequency of 50 Hz and 50 kHz in the multi- and single-channel experiments, respectively. Amplitude, lifetime, and fluctuation analysis was performed with ClampFit 10.2 (Molecular Devices) and OriginPro 8.5 (OriginLab) software as well as with software developed in house.

### Reproducibility of the experiments and statistics

All experiments were performed independently at least two times. The results from the representative experiments are shown in the figures. Values (*n* = 3) are calculated as mean ± standard deviation (S.D.) using the Prism4 Software (GraphPad Software, Inc., La Jolla, CA, USA). The values shown are the average of 3 trials and the error bars represent standard error of the mean. Significance was tested by using the student's t-test (***p<0.0005; ** p<0.005; *p<0.05; n.s. = not significant).
